# COVID-19 in pregnant women: An evaluation of clinical symptoms and laboratory parameters based on the 3 trimesters

**DOI:** 10.15537/smj.2022.43.4.20210904

**Published:** 2022-04

**Authors:** Şeyhmus Tunç, Mehmet Rifat Göklü, Süleyman Cemil Oğlak

**Affiliations:** *From the Department of Obstetrics and Gynecology, Health Sciences University, Gazi Yaşargil Training and Research Hospital, Diyarbakır, Turkey.*

**Keywords:** COVID-19, pregnancy, pregnancy trimesters

## Abstract

**Objectives:**

To investigate the association between the hospitalization rates, symptoms, and laboratory parameters of pregnant women diagnosed with coronavirus disease 2019 (COVID-19) and the gestational week, and determine their symptoms or laboratory parameters predictive of the need for possible admission in the intensive care unit (ICU).

**Methods:**

We retrospectively analyzed the symptoms, laboratory parameters, and treatment modalities of 175 pregnant women with COVID-19 who were admitted to a tertiary referral hospital between March 2020 and March 2021 and investigated their association with pregnancy trimesters.

**Results:**

The COVID-19-related hospitalization rates in the first trimester was 24.1%, second trimesters was 36%, and third trimester was 57.3%. Cough and shortness of breath were significantly higher in the pregnant women in their third trimester than those in the first 2 trimesters (*p*=0.042 and *p*=0.026, respectively). No significant relationship was found between pregnancy trimesters and the need for ICU admission. Shortness of breath at the first admission increased the need for ICU by 6.95 times, and a 1 unit increase in C-reactive protein (CRP) level increased the risk of ICU by 1.003 times.

**Conclusion:**

The presence of respiratory symptoms and the need for hospitalization increased significantly with later trimesters in pregnant women with COVID-19. The presence of shortness of breath or high CRP level at the time of admission could predict the need for ICU admission.

**T**he first case of pneumonia of an unknown origin was identified in Wuhan, the capital of Hubei province, China, in December 2019.^1,2^ The World Health Organization defined this disease caused by severe acute respiratory syndrome coronavirus 2 (SARS-CoV-2) as coronavirus disease 2019 (COVID-19) and declared a pandemic in 2020 as a result of its rapid spread.^3,4^ As of September 2021, the total number of recorded cases of Coronavirus disease 2019 worldwide has approached 250 million and that of deaths has exceeded 4.5 million.^5^

Coronavirus disease 2019 mainly manifests as a pulmonary disease with flu-like symptoms such as fever, cough, shortness of breath, fatigue, and headache.^6^ Severe acute respiratory syndrome coronavirus 2 infection may be asymptomatic or cause critical illness that canresult in pneumonia and respiratory failure.^7^ Neurological, renal, hepatic, gastrointestinal, thromboembolic, cardiac, endocrine, and dermatological symptoms may be present during the disease course.^8^

Pregnancy is a unique immunological situation that is modulated since the immune system is affected by signals generating from the placenta.^9-14^ Previous studies indicated compelling evidence that pregnant women are at a greater risk of severe disease and mortality from viral infections, notably during pandemics and particularly those involved in the respiratory system.^9^ During pregnancy, a 9.5-25% decrease occurs in the functional residual capacity. Moreover, oxygen use increases by 21% due to physiological hyperventilation.^15^ Also, changes in the nasal mucosa induced by progesterone tend to facilitate the retention of the virus in the upper respiratory tract, thus making it difficult for the host immunity to remove it.^16^ The disease causes destruction, inflammation, and hemorrhage in type 1 and 2 pneumocytes through the angiotensin-converting enzyme-2 (ACE-2) receptor in the lungs.^17,18^ Compared with nonpregnant women, pregnant women show a 2-fold increase in the expression of ACE-2 receptors.^19^ Thus, pregnant women are at a higher risk of severe illness and death than nonpregnant women; the former group also has a higher need for admission in the intensive care unit (ICU) and oxygen support than the latter.^20^

Coronavirus disease 2019 in pregnant women is a popular research topic. The present study aimed to investigate the association between the hospitalization rates, symptoms, and laboratory parameters of pregnant women diagnosed with COVID-19 and the week of gestation and determine the symptoms or laboratory parameters that can predict the need for possible ICU admission.

## Methods

This retrospective study examined the clinical and laboratory findings and treatment modalities of 175 pregnant women diagnosed with COVID-19 who were admitted Diyarbakır Gazi Yaşargil Training and Research Hospital, Turkey, which was a tertiary referral hospital, between March 2020 and March 2021. Ethical approval for the study was obtained from the hospital’s ethical committee. The study was carried out in accordance with the Declaration of Helsinki. This study included pregnant women diagnosed with COVID-19 and followed up at our hospital (outpatient or inpatient).Patient data were retrieved from the hospital’s archive system and patient files.

Severe acute respiratory syndrome coronavirus 2 nucleic acid was detected in all pregnant women by real-time upper respiratory tract specimen polymerase chain reaction (PCR). Pregnant women with COVID-19 experienced clinical evaluation of vital signs, laboratory analysis, and radiologic chest assessment at admission. A chest x-ray or computed tomography (CT) was performed for pneumonia diagnosis. All patients signed the informed consent before chest x-ray (n=175) and CT examination (n=56). During the x-ray and CT examination their pelvis and lower abdomen were covered with a lead blanket. Each participant in the study cohort was an independent sample. All participants have enrolled in the study according to their findings at the time of admission (specimen date).

Based on the national COVID-19 guideline of the Turkish Ministry of Health, pregnant women with pneumonia were divided into 2 groups, namely, mild-moderate (respiratory rate <30/minute (min); SpO2 level >90% in ambient air; and bilateral diffuse, <50% lung involvement on imaging) and severe (fever, muscle/joint pains, cough, and sore throat; tachypnea ≥30/min; SpO2 level ≤90% in ambient air; and bilateral diffuse pneumonia findings on chest x-ray or tomography) disease.^21^ Pregnant women with mild-moderate pneumonia who did not need oxygen therapy were followed up as outpatients. Pregnant women with mild to moderate pneumonia requiring oxygen therapy and those with severe pneumonia were hospitalized.

Clinical and laboratory parameters obtained at the first admission were used in our study. The clinical parameters were examined in terms of fever, cough, shortness of breath, headache, loss of smell, diarrhea, and myalgia. The laboratory parameters were examined in terms of white blood cell (WBC) counts, lymphocyte count, neutrophil count, hemoglobin, hematocrit, platelet, glucose, urea, creatinine, aspartate aminotransferase (AST), alanine aminotransferase (ALT), lactate dehydrogenase (LDH), D-dimer, C-reactive protein (CRP), ferritin, and procalcitonin.

Owing to the risk of influencing the clinical and laboratory parameters, COVID-19-positive pregnant women with comorbidities such as diabetes, hypertension, coronary artery disease, and asthma as well as those hospitalized because of obstetric reasons were not included in this study.

### Statistical analysis

The data analysis was performed using the Statistical Package for the Social Sciences, version 26 (IBM Corp., Chicago, IL, USA). The data used here were tested for violations of assumptions of parametric tests, such as using the Levene test for homogeneity of variances and Shapiro Wilk test with Q-Q plots for normality. So, to identify differences between independent 2 groups, Mann-Whitney U or Student’s t-test were used. For the continuous variables which were compared among more than 2 independent groups; Kruskal Wallis or One Way ANOVA test was used with post hoc multi comparison tests to identify the groups that make the difference. The Chi-square or Fisher’s exact test were used to compare groups among the categories of variables. Data were presented as mean ± standard deviation and (median-range) values and as numbers with relevant percentages. To define risk factors of outcome variables, multiple logistic regression analysis and adjusted odds ratios with their confidence intervals were calculated. All covariates with missing data in less than 20% of observations and a *p*-value of <0.05 in univariate testing were considered for inclusion in the final multiple regression model and retained if the *p*-value was <0.05. Highly collinear covariates (defined as correlation coefficient >0.5) were not included together in the final multivariate model. The goodness of model fit was assessed by Hosmer-Lemeshow test. Whether the CRP variable has diagnostic power to determine ICU need, recdeiver operating characteristic analysis was used and the Youden index was calculated to determine cut-off value. A *p*-value of <0.05 was considered statistically significant for all statistical processes.

## Results

In our study cohort, COVID-19 patients were not vaccinated against the SARS-CoV-2. The hospitalization rate was 45.7% after including all pregnant women in the study. This rate increased in later gestational weeks. Outpatient follow-up was proportionally higher in patients in their first and second trimesters. The hospitalization rate in patients in their third trimester was higher than that in the outpatient groups. Cough was the most common (68%) symptom among the cohort. Other symptoms included high fever (48.5%), shortness of breath (48.5%), headache (33%), myalgia (32%), diarrhea (10%), and the loss of smell and taste (8%). Comparisons of clinical symptoms among the groups based on trimester indicated that compared with the patients in their first 2 trimesters, the presence of cough and shortness of breath due to COVID-19 was significantly higher in those in their third trimester (Table 1).

**Table 1:** - Comparisons of the clinical symptoms among the trimester groups.

Symptoms	First trimester				Second trimester			Third trimester			*P*-value
		n	Row (%)	Column (%)	n	Row (%)	Column, (%)	n	Row (%)	Column (%)	
Outpatient follow-up		22	23.2	75.9	32	33.7	64.00	41	43.2	42.7	**0.002**
Hospitalization		7	8.6	24.1	18	22.5	36.0	55	68.8	57.3	**0.002**
Fever	−	15	16.8	51.7	28	31.1	56.0	47	52.2	49.0	0.721
	+	14	16.5	48.3	22	25.9	44.0	49	57.7	51.0	
Cough	−	12	21.4	41.4	21	37.5	42.0	23	41.1	24.0	**0.042**
	+	17	14.3	58.6	29	24.4	58.0	73	61.3	76.0	
Shortness of breath	−	16	17.8	55.2	33	36.7	66.0	41	45.6	42.7	**0.026**
	+	13	15.3	44.8	17	20.0	34.0	55	64.7	57.3	
Headache	−	17	14.5	58.6	30	25.6	60.0	70	59.8	72.9	0.187
	+	12	20.7	41.4	20	34.7	40.0	26	44.8	27.1	
Loss of smell and taste	−	28	17.4	96.6	45	27.9	90.0	88	54.7	91.7	0.640*
	+	1	7.1	3.5	5	35.7	10.0	8	57.1	8.3	
Diarrhea	−	26	16.5	89.7	44	27.9	88.0	88	55.7	91.7	0.731*
	+	3	17.7	10.3	6	35.3	12.0	8	47.1	8.3	
Myalgia	−	15	12.6	51.7	38	31.9	76.0	66	55.5	68.8	0.081
	+	14	25.0	48.3	12	21.4	24.0	30	53.6	31.3	
Intensive care unit admission	−	29	17.3	100.0	47	28.0	94.0	92	54.8	95.8	0.432*
	+	0	0.0	0.0	3	42.9	6.0	4	57.1	4.2	
Severe illness	−	29	17.6	100.0	46	27.9	92.0	90	54.5	93.8	0.369*
	+	0	0.0	0.0	4	40.0	8.0	6	60.0	6.3	

*Indicates *p*-values obtained from Fisher’s exact test. Bold values denote statistical significance at the *p*<0.05 level.

The evaluation of the selected blood parameters in the cohort indicated that 56.5% had lymphocytopenia, 35.4% had an elevated CRP level, 32% had an elevated LDH level, 30% had an elevated ALT level, 24% had an elevated D-dimer level, 22% had leukocytosis, and 12.5% had an elevated AST level. Comparisons among the trimester groups based on laboratory parameters also indicated that the leukocyte count increased in all 3 trimesters and the rate of increase was the highest in the third trimester. The decrease in lymphocyte counts was observed in all 3 trimesters and lymphocytopenia intensified in the later trimester. Varying degrees of increase in the serum levels of AST, ALT, LDH, D-dimer, and CRP were detected with progressing trimester (Table 2).

**Table 2:** - Comparisons of the laboratory finding abnormalities among the trimester groups.

Laboratory findings (reference ranges)*	First trimester (n=29)	Second trimester (n=50)	Third trimester (n =96)
***Leucocytes (10*** ^***3***^ ***/mm*** ^*3*^ ***reference range)***	(5.7–13.6)	(5.6–14.8)	(5.9–16.9)
Increased	6 (21)	7 (14)	26 (27)
Decreased	2 (7)	-	-
***Lymphocytes (%; reference range)***	(19–26)	(16–26)	(16–21)
Increased	1 (4)	-	-
Decreased	11 (37)	28 (56)	60 (62)
***Aspartate transaminase (U/L; reference range)***	(3–23)	(3–33)	(4–32)
Increased	-	7 (14)	15 (16)
***Alanine transaminase (U/L; reference range)***	(3–30)	(2–33)	(2–25)
Increased	1 (4)	6 (12)	7 (7)
***Lactate dehydrogenase (U/L; reference range)***	(78–433)	(80–447)	(82–524)
Increased	4(14)	14(28)	38 (40)
***D-dimer (μg/L; reference range)***	(500–950)	(320–1290)	(130–1700)
Increased	-	4 (8)	38 (34)
***C-reactive protein (mg/L; reference range)***	(0.2–3.0)	(0.4–20.3)	(0.4–8.1)
Increased	9 (31)	11 (22)	42 (44)

Values are presented as number and percentages (%).

Comparisons among the trimester groups in terms of laboratory parameters indicated that D-dimer and procalciton in levels varied across trimesters. A significant increase was noted in the median value of the D-dimer level with progressing trimester. Although the median values of the procalciton in level were similar in the first 2 trimesters, these were significantly higher in the third trimester. The medians that accounted for the difference are explained using letter indices in Table 3.

**Table 3:** - Comparisons of the clinical symptoms and laboratory parameters among the trimester groups

	First trimester		Second trimester		Third trimester		*P*-value
	Mean ± SD	Median–Range	Mean ± SD	Median–Range	Mean ± SD	Median–Range	
Age	32.43 ± 5.74	34–17	31 ± 5.81	30.5–20	29.81 ± 6.66	28–29	0.526*
White blood cells	6.27 ± 1.38	6.3–3.8	7.51 ± 2.58	6.95–7.9	8.96 ± 3.33	7.9–13.1	0.444
Lymphocyte%	25.03 ± 6.19	24.3–16.4	20.69 ± 7.49	19.55–27.2	18.89 ± 6.8	17.8–29.5	0.079*
Neutrophil%	65.5 ± 7.94	66.1–21.4	72.97 ± 8.34	73.75–30.2	75.03 ± 7.71	75–34.2	0.096
Hemoglobin	12.39 ± 1.77	13.3–4.1	11.87 ± 1.26	12.3–5	11.17 ± 1.65	11.2–7.7	0.093
Hematocrit	38.64 ± 4.38	40.6–10.8	36.84 ± 3.36	38.15–12.6	35.49 ± 4.53	35.3–22.2	0.093
Platelet	229.29 ± 40.33	230–118	243.11 ± 93.17	215.5–399	236.98 ± 69.64	238–353	0.476
Glucose	82.5 ± 10.32	79–26	94.06 ± 30.44	87–134	85.13 ± 24.36	77–119	0.288
Creatinine	0.59 ± 0.09	0.6–0.2	0.49 ± 0.07	0.5–0.3	0.53 ± 0.08	0.5–0.3	0.072
Aspartate transaminase	19.71 ± 7.52	20–24	44.29 ± 60.57	20–194	36 ± 53.05	22–356	0.749
Alanine transaminase	19.43 ± 13.94	14–39	38.65 ± 57.27	16–204	27.76 ± 55	15–401	0.535
Lactate dehydrogenase	209.67 ± 51	213–124	222.76 ± 57.67	219–237	266.67 ± 104.59	246–470	0.512
D-dimer	211.2 ± 70.34	197–187^a^	288.94 ± 123.2	268.5–404^b^	953.9 ± 1202.71	525–6213^c^	**<0.001**
C-reactive protein	18.33 ± 18.9	11–42.5	26.02 ± 33.76	12.9–130	23.98 ± 30.91	11.8–119	0.962
Ferritin	75.5 ± 91.1	24.5–211	105.44 ± 105.55	81–431	66.7 ± 91.94	27.5–452	0.068
Procalcitonin	0.04 ± 0.03	0.02–0.07^a^	0.11 ± 0.21	0.04–0.76^a^	0.13 ± 0.18	0.07–0.9^b^	**0.020**

*Indicates *p*-values obtained from analysis of variance, whereas all other *p*-values were obtained from Kruskal-Wallis test. a,b,c indicate that medians with same indices are the same and the others are statistically different from one another. Bold values denote statistical significance at the *p*<0.05 level. SD: standard deviation

Of the cohort, 5.7% (n=10) had severe disease. Among the pregnant women diagnosed with COVID-19, 70% (n=7) with severe disease needed ICU admission. No significant difference was observed in the rates of ICU admission due to COVID-19 among the trimester groups. The presence of dyspnea was significantly high in patients who needed ICU admission regardless of their trimester (Table 4).

**Table 4:** - Comparisons of the laboratory parameters among patients admitted in the intensive care unit.

Parameters		ICU admission (−)		ICU admission (+)		P-value
		Row (%)	Column (%)	Row (%)	Column (%)	
Trimester	First	100.0	17.26	0.0	0.0	0.432*
	Second	94.0	27.98	6.0	42.9	
	Third	95.8	54.76	4.2	57.1	
Fever	−	94.4	50.60	5.2	71.4	0.445*
	+	97.6	49.40	2.4	28.6	
Cough	−	98.2	32.70	1.8	14.3	0,432*
	+	95.0	67.30	5.0	85.7	
Shortness of breath	−	100.0	53.60	0.0	0.0	0.006*
	+	91.8	46.40	8.2	100.0	
Headache	−	94.0	65.50	6.0	100.0	0.097*
	+	100.0	34.50	0.0	0.0	
Loss of smell and taste	−	95.7	91.70	4.3	100.0	0.426
	+	100.0	8.30	0.0	0.0	
Diarrhea	−	95.6	89.90	4.4	100.0	0.376
	+	100.0	10.10	0.0	0.0	
Myalgia	−	94.1	66.70	5.9	100.0	0.098*
	+	100.0	33.30	0.0	0.0	

*Indicates *p*-values obtained from Fisher’s exact test. Bold values denote statistical significance at the *p*<0.05 level. ICU: intensive care unit

Independent of the trimester, CRP and procalciton in levels were significantly higher in patients who needed ICU admission (Table 5).

**Table 5:** - Comparisons of the laboratory parameters among patients admitted in the intensive care unit.

Characteristics	ICU admission (−)		ICU admission (+)		*P*-value
	Mean ± SD	Median–Range	Mean ± SD	Median–Range	
Age, years	28.89 ± 6.06	28–30	32.43 ± 7.46	34–22	0.154*
Gestational week at admission	25.4 ± 10.05	27–34	29.43 ± 8.94	33–23	0.284*
White blood cells	8.57 ± 3.16	7.9–14.1	6.83 ± 2.47	5.8–6.8	0.084
Lymphocyte	19.49 ± 7.78	18.4–43.6	19.86 ± 6.06	20.7–16.7	0.810*
Neutrophil	73.23 ± 10.21	74.2–80.19	74.81 ± 7.06	72.7–20.2	0.818*
Hemoglobin	11.54 ± 1.55	11.7–7.8	11.49 ± 1.15	11.5–3	0.887
Hematocrit	39.27 ± 33.51	36.5–378.5	35.83 ± 3.23	36.2–8.9	0.698
Platelet	237.02 ± 71.28	230–477	224 ± 34.26	230–96	0.722
Glucose	90.95 ± 28.13	84–201	106 ± 29.92	108–79	0.155
Urea	14.07 ± 5.22	13–26	15.29 ± 4.96	16–12	0.476*
Creatinine	0.52 ± 0.08	0.5–0.4	0.54 ± 0.05	0.5–0.1	0.351*
Aspartate transaminase	28.26 ± 41.4	19–359	56.29 ± 68.09	34–192	0.064
Alanine transaminase	23.65 ± 41.8	15–401	44.29 ± 73.84	16–203	0.439
Lactate dehydrogenase	245.19 ± 94.83	224.5–484	295 ± 81.17	300–228	0.070
D-dimer	736.84 ± 1049.08	413.5–6277	769.57 ± 986.89	411–2763	0.939
C-reactive protein	18.4 ± 24.4	11.2–130	62.3 ± 46.69	89.3–107.2	**0.010**
Ferritin	66.99 ± 89.2	30–452	137.86 ± 113.5	137–321	0.081
Procalcitonin	0.11 ± 0.18	0.05–0.9	0.2 ± 0.15	0.21–0.42	**0.041**
Vitamin D	15.25 ± 9.48	12.55–32.2	18.2 ± 5.94	18.2–8.4	0.732

*Indicates the *p*-values obtained from student’s t-test, whereas all other *p*-values were obtained from Mann–Whitney U test. Bold values denote statistical significance at the *p*<0.05 level. ICU: intensive care unit, SD: Standard deviation

After adjusting the variables, including maternal age and gestational week, multiple logistic regression analysis was performed using the clinical symptoms and laboratory parameters of the pregnant women who needed ICU admission at the time of hospitalization. Table 6 presents the results obtained based on the variables of shortness of breath, and CRP level, which were significant. The presence of shortness of breath increased the need for ICU admission by 6.95 times, and an increase of 1 unit in the level of CRP increased the risk of ICU admission by 1.003 times.

**Table 6:** - Multiple regression analysis of parameters associated with ICU need.

	Beta coefficient	Standard error	*P*-value	OR	95% CI for OR	
					Lower	Upper
Constant	−4.359	1.205	**0.000**	0.013		
C-reactive protein	0.031	0.012	**0.009**	1.031	1.008	1.056
Shortness of breath (+)	1.939	1.081	0.073	6.949	0.835	57.841

Bold values denote statistical significance at the *p*<0.05 level. OR: odds ratio, CI: confidence interval

**Figure 1:**
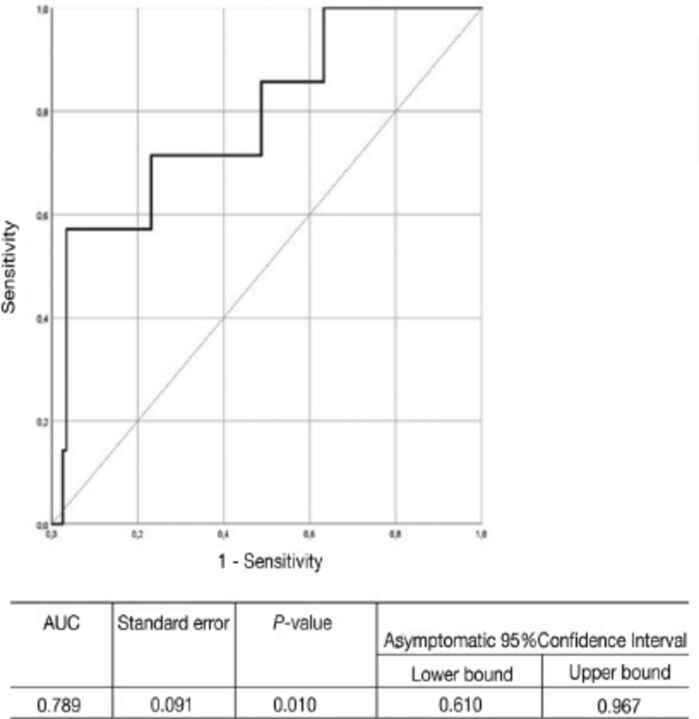
- Receiver operating characteristic curve for serum C-reactive protein value for predicting the requirement of intensive care unit. AUC: under area the curve

As a result of the ROC analysis (Figure 1), it was understood that the CRP variable is a parameter that can be used to determine ICU admission (*p*=0.010). For this case, the cut-off value calculated according to the Youden index was found to be 77.2 mg/dL (sensitivity=57.1%, and specificity=96.6%).

## Discussion

The present study revealed that the need for ICU admission due to COVID-19 increased with progressing trimester. Although the majority of the pregnant women in their first 2 trimesters were followed-up as outpatients, the majority of those in their third trimester were followed-up as inpatients. A significant increase was observed in the cough and shortness of breath symptoms in the pregnant women in their third trimester. However, no significant difference was detected in the laboratory parameters among the trimester groups. Also, the gestational week did not have any significant effect on the need for ICU admission. Shortness of breath and high CRP levels at the time of admission was significantly associated with the need for subsequent ICU admission.

According to Centers for Disease Control and Prevention data of January-June 2020, 31.5% of pregnant women were hospitalized for COVID-19. The reported rate was higher than that reported in nonpregnant women (5.8%).^22^ Zambrano et al^20^ reported that pregnant women with COVID-19 had an increased risk of severe disease, need for ICU admission, and mortality compared with nonpregnant women.The higher rate of hospitalization due to COVID-19 than the normal population might be associated with the increased load on the cardiopulmonary system and the suppressed immune system during pregnancy. In the present study, the rate of hospitalization during pregnancy was 45.7%, which was well above that reported in the literature and for the normal population. The hospitalization rates increase with the progressing gestational week. Of the patients in their third trimester, 68.7% needed hospitalization. This may be associated with the increase in oxygen demand with the gestational week, physiological hyperventilation, and aggravation of dyspnea.

Mohr-Sasson et al^23^ reported that cough was the most common symptom in 68% of pregnant women with COVID-19. Other symptoms included high fever in 48.5% of the pregnant women, shortness of breath in 48.5%, myalgia in 32%, diarrhea in 10%, and the loss of smell and taste in 8%. In our study, comparisons among the trimester groups indicated that the presence of respiratory symptoms (such, cough and shortness of breath) associated with COVID-19 was significantly higher among patients in their third trimester than those in their first 2 trimesters. As mentioned above, this can be explained by the fact that the physiological changes related to pregnancy become more pronounced with the progressing gestational week. These outcomes also led to an increase in hospitalization rates. Our study revealed no significant association between other symptoms and trimester.

In a meta-analysis by Diriba et al,^24^ 28.4% of the pregnant women with COVID-19had leukocytosis, 63% hadlymphocytopenia, and 55.9% of the patients had high CRP levels. Their study underlined lymphocytopenia as the most frequently reported laboratory finding. Mohr-Sasson et al^23^ reported the presence of lymphocytopenia in 45.5% of pregnant patients. The relative lymphocyte count to that of WBCs was significantly reduced in the pregnant group compared with the nonpregnant women (*p*=0.003). Consistent with the literature, in our study, lymphocytopenia was noted in 56.5% of patients, and an elevated CRP level in 35.4% of patients. According to a study on pregnant women with COVID-19, the need for ICU admission of the mother or fetus due to COVID-19 was 31.3% and the maternal mortality rate was 2.7%.^24^ In the present study, the rate of severe patients was 5.7%, and the rate of ICU admission was 4%. These values were consistent with the literature. There was no significant difference between the trimesters by the need for ICU admission.

A review of all groups included in our study indicated a significant association between CRP elevation at the time of admission or especially the complaint of shortness of breath and subsequent ICU admission. It was determined that the shortness of breath increased the risk of ICU admission by 6.95 times. Respiratory symptoms are the most common cause of COVID-19-related emergency department admissions in pregnancy.^23^ Pregnant women who have adapted to the physiological dyspnea secondary to cardiopulmonary changes can initially tolerate the increased oxygen demand due to COVID-19, and therefore, it may take time before the respiratory symptoms become evident. Based on the foregoing assumptions, it can be suggested that pregnant women present to the emergency department late and have a more severe disease at the time of admission than the normal population.

### Study limitations

This study has been designed retrospectively. Moreover, our study group comprised of pregnant women, and we did not include a control group in this study. Our study examined the parameters at the time of hospitalization and the laboratory parameters and clinical symptoms during the course of hospital stay were not included. The strength of our study is a relatively large sample size. Also,to the best of our knowledge, there are few studies in the literature examining the association between COVID-19 and pregnancy trimesters.

In conclusion, the symptoms of dyspnea and cough increased significantly and the need for hospitalization increased with the progressing gestational week. Pregnancy-related physiological changes may have worsened the symptoms of COVID-19. We found a significant association between shortness of breath and higher CRP level at the first admission and subsequent need for ICU admission regardless of trimester.

Therefore, we highly suggest that pregnant women be evaluated regarding shortness of breath and CRP levels at the first admission. We also recommend that pregnant women who are followed up at home because of asymptomatic or mild illness should be provided with detailed information about the symptoms, particularly shortness of breath, and that they should be closely monitored during the quarantine period, at least utilizing daily phone calls.

## References

[1] Huang C , Wang Y , Li X , Ren L , Zhao J , Hu Y , et al. Clinical features of patients infected with 2019 novel coronavirus in Wuhan, China. Lancet 2020; 395: 497–506. 31986264 10.1016/S0140-6736(20)30183-5 PMC7159299

[2] Oğlak SC , Obut M. The risk of vicarious trauma among front-line and non-front-line midwives and nurses: Vicarious traumatization among medical staff. Aegean J Obstet Gynecol 2020; 2: 1–4.

[3] World Health Organization. Timeline COVID-19. (Updated 2022; Accessed 2020 May 15]. Available form: https://www.who.int/news-room/detail/27-04-2020-who-timeline—covid-19

[4] Can E , Oğlak SC , Ölmez F. Abnormal liver function tests in pregnant patients with COVID-19 - a retrospective cohort study in a tertiary center. Ginekol Pol 2022; 93; 151–157. 10.5603/GP.a2021.0182 35072238

[5] Baj J , Karakuła-Juchnowicz H , Teresiński G , Buszewicz G , Ciesielka M , Sitarz E , et al. COVID-19: Specific and non-specific clinical manifestations and symptoms: The current state of knowledge. J Clin Med 2020; 9: 1753. 32516940 10.3390/jcm9061753 PMC7356953

[6] Moore KM , Suthar MS. Comprehensive analysis of COVID-19 during pregnancy. Biochem Biophys Res Commun 2021; 538: 180–186. 33384142 10.1016/j.bbrc.2020.12.064 PMC7759124

[7] Harrison AG , Lin T , Wang P. Mechanisms of SARS-CoV-2 transmission and pathogenesis. Trends Immunol 2020; 41:1100–1115. 33132005 10.1016/j.it.2020.10.004 PMC7556779

[8] Gupta A , Madhavan MV , Sehgal K , Nair N , Mahajan S , Sehrawat TS , et al. Extrapulmonary manifestations of COVID-19. Nat Med 2020; 26: 1017–1032. 32651579 10.1038/s41591-020-0968-3 PMC11972613

[9] Silasi M , Cardenas I , Kwon JY , Racicot K , Aldo P , Mor G. Viral infections during pregnancy. Am J Reprod Immunol 2015; 73: 199–213. 25582523 10.1111/aji.12355 PMC4610031

[10] Oğlak SC , Obut M. Expression of ADAMTS13 and PCNA in the placentas of gestational diabetic mothers. Int J Morphol 2021; 39: 38–44.

[11] Behram M , Oğlak SC , Doğan Y. Evaluation of BRD4 levels in patients with early-onset preeclampsia. J Gynecol Obstet Hum Reprod 2021; 50: 101963. 33129979 10.1016/j.jogoh.2020.101963

[12] Behram M , Oğlak SC , Dağ İ. Circulating levels of Elabela in pregnant women complicated with intrauterine growth restriction. J Gynecol Obstet Hum Reprod 2021; 50: 102127. 33781971 10.1016/j.jogoh.2021.102127

[13] Behram M , Oğlak SC. The expression of angiogenic protein Cyr61 significantly increases in the urine of early-onset preeclampsia patients. J Contemp Med 2021; 11: 605–609.

[14] Oğlak SC , Tunç Ş , Ölmez F. First trimester mean platelet volume, neutrophil to lymphocyte ratio, and platelet to lymphocyte ratio values are useful markers for predicting preeclampsia. Ochsner J 2021; 21: 364–370. 34984051 10.31486/toj.21.0026 PMC8675624

[15] LoMauro A , Aliverti A. Respiratory physiology of pregnancy: Physiology masterclass. Breathe (Sheff) 2015; 11: 297–301. 27066123 10.1183/20734735.008615 PMC4818213

[16] Vale AJM , Fernandes ACL , Guzen FP , Pinheiro FI , de Azevedo EP , Cobucci RN. Susceptibility to COVID-19 in pregnancy, labor, and postpartum period: Immune system, vertical transmission, and breastfeeding. Front Glob Womens Health 2021; 2: 602572. 34816177 10.3389/fgwh.2021.602572 PMC8593969

[17] Carsana L , Sonzogni A , Nasr A , Rossi RS , Pellegrinelli A , Zerbi P , et al. Pulmonary post-mortem findings in a series of COVID-19 cases from northern Italy: a two-centre descriptive study. Lancet Infect Dis 2020; 20: 1135–1140. 32526193 10.1016/S1473-3099(20)30434-5 PMC7279758

[18] Ackermann M , Verleden SE , Kuehnel M , Haverich A , Welte T , Laenger F , et al. Pulmonary vascular endothelialitis, thrombosis, and angiogenesis in Covid-19. N Engl J Med 2020; 383: 120–128. 32437596 10.1056/NEJMoa2015432 PMC7412750

[19] Brosnihan KB , Neves LA , Anton L , Joyner J , Valdes G , Merrill DC. Enhanced expression of Ang-(1-7) during pregnancy. Braz J Med Biol Res 2004; 37: 1255–1262. 15273828 10.1590/s0100-879x2004000800017

[20] Zambrano LD , Ellington S , Strid P , Galang RR , Oduyebo T , Tong VT , et al. Update: Characteristics of Symptomatic Women of Reproductive Age with Laboratory-Confirmed SARS-CoV-2 Infection by Pregnancy Status - United States, January 22-October 3, 2020. MMWR Morb Mortal Wkly Rep 2020; 69: 1641–1647. 33151921 10.15585/mmwr.mm6944e3 PMC7643892

[21] T.C. Sağlık Bakanlığı COVID-19 Bilgilendirme Platformu.Republic of Turkey Ministry of Health. COVID-19 Information Platform [Updated 2022; Accessed 2020 June 4]. Available from: https://covid19.saglik.gov.tr/Eklenti/41940/0/covid19-toplumdasalginyonetimirehberi-19112021pdf.pdf

[22] Ellington S , Strid P , Tong VT , Woodworth K , Galang RR , Zambrano LD , et al. Characteristics of women of reproductive age with laboratory-confirmed SARS-CoV-2 infection by pregnancy status - United States, January 22-June 7, 2020. MMWR Morb Mortal Wkly Rep 2020; 69: 769–775. 32584795 10.15585/mmwr.mm6925a1 PMC7316319

[23] Mohr-Sasson A , Chayo J , Bart Y , Meyer R , Sivan E , Mazaki-Tovi S , et al. Laboratory characteristics of pregnant compared to non-pregnant women infected with SARS-CoV-2. Arch Gynecol Obstet 2020; 302: 1–6. 32572616 10.1007/s00404-020-05655-7 PMC7307945

[24] Diriba K , Awulachew E , Getu E. The effect of coronavirus infection (SARS-CoV-2, MERS-CoV, and SARS-CoV) during pregnancy and the possibility of vertical maternal-fetal transmission: a systematic review and meta-analysis. Eur J Med Res 2020; 25: 39. 32887660 10.1186/s40001-020-00439-w PMC7471638

